# Supplementary material for: The Effect of PCL Addition on 3D-Printable PLA/HA Composite Filaments for the Treatment of Bone Defects
Source: Polymers (Basel). 2022 Aug 13;14(16):3305. doi: 10.3390/polym14163305 (PMC9416491; doi:10.3390/polym14163305)
Supplement: Supplementary file 1 [file polymers-14-03305-s001.zip › polymers-1839574-supplementary.pdf]

polymers

MDPI

Supplementary materials

The Effect of PCL Addition on 3D-Printable PLA/HA Composite Filaments for the Treatment of Bone Defects

Elin Åkerlund [1], Anna Diez-Escudero [2,*], Ana Grzeszczak [1] and Cecilia Persson [1,*]

[1] Division of Biomedical Engineering, Department of Materials Science and Engineering, The Ångström Laboratory, Uppsala University, Uppsala, Sweden;
[2] Ortholab, Department of Surgical Sciences, Rudbeck laboratory, Uppsala University, Uppsala, Sweden;
* Correspondence: cecilia.persson@angstrom.uu.se; anna.diez@surgsci.uu.se

Supplementary Materials

Table S1. Extrusion settings for each material.

Sample	Temperatures (°C)				Speed (rpm)	Fan speed (%)
	Heater 4	Heater 3	Heater 2	Heater 1		
90PLA10PCL	170	185	190	180	5	70
80PLA20PCL	170	185	190	180	5	60
70PLA30PCL	140	155	160	160	6	60
90PLA10PCL-15HA	170	185	180	175	5	70
80PLA20PCL-15HA	170	185	190	180	5	70
70PLA30PCL-15HA	140	155	160	160	6	60

Table S2. Thermal characteristics of all pristine samples investigated (before degradation) illustrating the glass transition temperature (T_g), the cold crystallization temperature (T_{cc}), the melting temperature (T_m).

Sample	Temperature (°C)		
	T_g	T_{cc}	T_m
PLA	59.9	133.7	152.6
PCL	-	-	55.4
90PLA10PCL	52.5	121.2	147.6
80PLA20PCL	52.7	115.0	146.0
70PLA30PCL	53.3	116.4	145.7
90PLA10PCL-15HA	52.9	114.9	146.4
80PLA20PCL-15HA	53.5	111.7	144.8
70PLA30PCL-15HA	54.5	122.6	146.3
